# The interplay between p16 serine phosphorylation and arginine methylation determines its function in modulating cellular apoptosis and senescence

**DOI:** 10.1038/srep41390

**Published:** 2017-01-25

**Authors:** Yang Lu, Wenlong Ma, Zhongwei Li, Jun Lu, Xiuli Wang

**Affiliations:** 1 The Institute of Genetics and Cytology, Northeast Normal University, Changchun 130024, P. R. China; 2 School of Life Sciences, Northeast Normal University, Changchun 130024, P. R. China

## Abstract

Cyclin-dependent kinase inhibitor p16^INK4a^ (p16) primarily functions as a negative regulator of the retinoblastoma protein (Rb) -E2F pathway, thus plays critical role in cell cycle progression, cellular senescence and apoptosis. In this study, we showed that the methylation of Arg 138 and the phosphorylation of Ser 140 on p16 were critical for the control of cell proliferation and apoptosis. Compared to wild type p16, mutant p16R138K possessed improved function in preventing cell proliferation and inducing apoptosis, while the Ser 140 mutation (p16S140A) exhibited the opposite alteration. We also demonstrated that H_2_O_2_ was able to induce the phosphorylation of p16, which facilitated the interaction between CDK4 (Cyclin-dependent protein kinase) and p16, in 293T (human emborynic kidney) cells. Furthermore, the elevated arginine methylation in p16S140A mutant and increased serine phosphorylation in p16R138K mutant suggest that a antagonizing mechanism coordinating Arg 138 methylation and Ser 140 phosphorylation to regulates p16 function as well as cellular apoptosis and senescence. These findings will therefore contribute to therapeutic treatment for p16-related gene therapy by providing theoretical and experimental evidence.

The p16 protein inhibits the activity of cyclin-dependent protein kinases (CDK4 and CDK6) to prevent the phosphorylation of the retinoblastoma protein (Rb) and subsequently lead to the accumulation of the hypophosphorylated form of Rb that mediates cell-cycle arrest^1^. Studies have shown that abnormal expression of p16 is frequently associated with different types of human tumors, such as melanoma, non-small cell lung carcinoma, breast cancer, colorectal cancer, bladder cancer and squamous cell carcinoma of the head and neck^2^,^3^,^4^.

Multiple mechanisms, including transcriptional regulation, DNA mutation and promoter methylation, are involved in the control of p16 expression. It has been demonstrated that various transcription factors, such as Ets and Bmi1, play critical roles in the transcriptional regulation p16 gene^5^. Our earlier studies showed that the histone acetyltransferase p300 recruited by Sp1 stimulated p16 transcription through inducing the H4 hyperacetylation on p16 gene, whereas HDAC3/4 inhibited the p16 promoter activity via the transcription factors YY1 and ZBP-89^6^,^7^,^8^.

Interestingly, recent work found that p16 phosphorylation increased in senescent human prostatic epithelial cells^9^. Subsequently, four phosphorylation sites, including Ser 7, Ser 8, Ser 140 and Ser 152, on p16 protein have been identified in human fibroblast cells, among these sites, only phosphorylated Ser 152 was detected in CDK4/6-bound p16^10^. In addition, another report showed that p16 phosphorylation at Ser 8 catalyzed by IKKβ relieved p16-mediated CDK4 inhibition^11^. Recently, we have demonstrated that p16 are methylated at Arg 22, Arg 131 and Arg 138 by PRMT6 and these modifications alleviated the cell cycle arrest at G1 via reducing p16-CDK4 interaction^12^. These evidence suggests important roles of post-translational modifications (PTMs) in the regulation of p16 function and cell fate.

A previous report showed that the arginine residues of FOXO1 was methylated by PRMT1, and this modification blocked the Akt-mediated phosphorylation of serine sites of the protein^13^. Moreover, PRMT1-mediated methylation of two arginine residues (Arg 94 and Arg 96) inhibited Akt-mediated phosphorylation of BAD at Ser 99 *in vitro* and *in vivo*^14^. It has also been shown that methylated Arg 1175 of EGFR mediated by PRMT5 positively modulates the EGF-induced EGFR trans-autophosphorylation at Tyr 1173^15^. These studies suggest that the interplays between arginine methylation and serine phosphorylation, especially at the neighboring positions, may be crucial to the functions of the proteins. Although p16 bears phosphorylated serines and/or methylated arginines, the crosstalk between these PTMs and its cellular functions still remain elusive.

In this study, we showed that the phosphorylation of p16 accumulated upon H_2_O_2_ in 293T cells. The phosphorylated forms of p16 gained increased binding affinity to CDK4 and appeared to be prone to protein degradation. Moreover, our data demonstrated that the level of arginine methylation on p16 increased as Ser 140 was replaced by alanine. Compare to wild type p16, this mutant led to alleviated cell cycle arrest and apoptosis. In contrast, the upregulated serine phosphorylation in p16R138K mutant was associated with enhanced cell cycle arrest and apoptosis. Taken together, our data outline a model where antagonistic crosstalk between Ser 140 phosphorylation and Arg 138 methylation orchestrates p16 to affect cell proliferation and apoptosis.

## Results

### Mutations of serines and arginines on p16 have different impacts on cell proliferation and apoptosis

**Figure 1:**
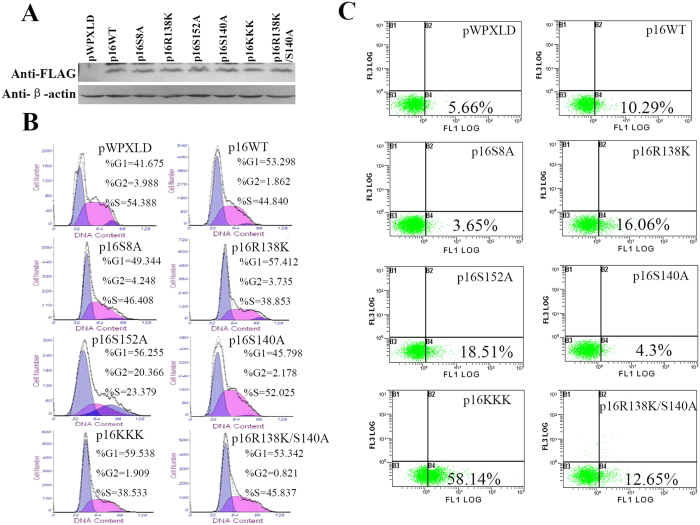
The serine and arginine site mutants of p16 protein exhibited different functions in cell proliferation and apoptosis. (**A**) Western blotting analysis of the p16 protein in 293T cells transfected with wild type p16 or mutant p16 expression plasmids, or empty control vector (pWPXLD) as a control. Flow cytometric analysis of cell cycle changes (**B**) and the apoptosis (**C**) after transfection of 293T cells with empty control vector (pWPXLD), wild type p16 or mutant p16 expression plasmids. (**B**) Cells were harvested at 48 h after transfection and stained with PI, analyzed by flow cytometry. (**C**) Cell apoptosis was measured by flow cytometry after annexin V and propidium iodide (PI) double staining after transfection 48 h.

A previous study demonstrated that p16 was phosphorylated at Ser 7, Ser 8, Ser 140 and Ser 152 in human fibroblast cells^10^. It seemed that these phosphorylation sites influenced p16 function differently^10^,^11^. Moreover, our previous study showed that methylation at Arg 22, Arg 131 and Arg 138 inhibited the activity of p16 to relieve the cell cycle arrest at G1 phase^12^. However, whether serine phosphorylation is coordinated with arginine methylation to control p16 function has not been characterized. To gain more insights into this issue, we mutated serine to alanine at different sites, including p16S8, p16S140 and p16S152 using pWPXLD-p16 expression plasmid. Subsequently, these mutants along with previously generated p16R138K, p16R22K/R131K/R138K (p16KKK) and p16R138K/S140A were expressed with comparable levels in 293T cells (Fig. 1A). As expected, the overexpression of wild type p16 caused an accumulation of cells in G1 phase (53.298%) as compared to the control (41.675%). Interestingly, the R138 mutant (p16R138K) and the triple arginine mutant (p16KKK) increased the population of G1 cells to 57.412% and 59.538%, while p16S8A and p16S140A had the opposite effect resulting in 49.344% and 45.796% G1 cells respectively (Fig. 1B). Notably, cells transfected with double mutant p16S140A/R138K displayed a similar G1 population (53.3%) to that of the wild type p16 (53.29%). It argues that the Arg 138 and Ser 140 of p16 may possess completely different functions in regulating p16 activity and the cell cycle. Moreover, the arginine mutants of p16 enhanced apoptosis as compared to wild type p16 in 293T cells. Under the same condition, p16 serine mutants reduced the apoptosis (Fig. 1C). Together, these results demonstrate that the arginine mutants (p16R138K and p16KKK) and serine mutant (p16S140A) of p16 have the opposite effect on cell cycle arrest and apoptosis.

### H_2_O_2_ enhances the phosphorylation of p16 to promote apoptosis

**Figure 2:**
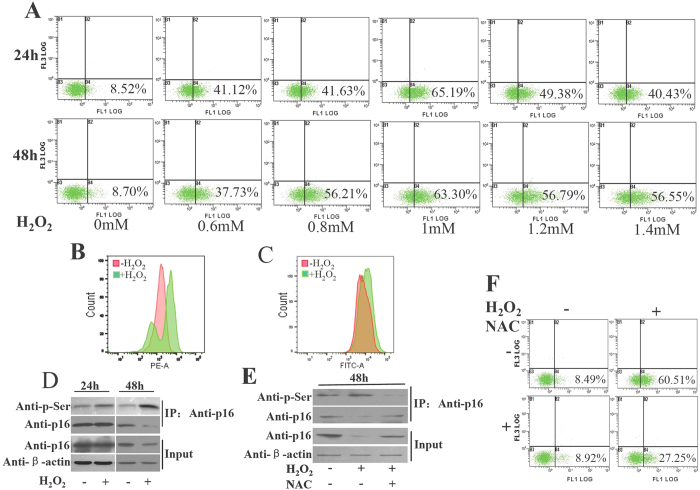
H_2_O_2_ enhanced the phosphorylation of p16 protein to promote apoptosis in 293T cells. (**A**) H_2_O_2_ induced apoptosis in 293T cells. 293T cells were treated with different concentration of H_2_O_2_ (0.6 mM, 0.8 mM, 1 mM, 1.2 mM or 1.4 mM) for 24 h or 48 h, and apoptosis was evaluated by flow cytometry. Apoptosis rates were calculated on the basis of 15 000 cells. (**B**,**C**) The 293T cells were treated 1 mM H_2_O_2_ for 24 h and then detected by ROS in mitochondria (**B**) and mitochondria membrane potential (**C**). (**D**) Immunoblots showing that the H_2_O_2_-induced serine phosphorylation of p16. Cell extracts were prepared and precipitated with anti-p16 antibody, then detected with anti-p16 antibody or anti-phosphserine antibody. (**E**) 293T cell extracts were prepared and precipitated with anti-p16 antibody, then detected in immunoblotting with anti-p16 antibody or anti-phosphserine antibody. Cells were treated with H_2_O_2_ or NAC and H_2_O_2_. Upper panel: samples co-immunoprecipitated with antibodies; Lower panel: proteins prior to immunoprecipitation (input). (**F**) Cells were treated with H_2_O_2_ or NAC, or a combination of both (cells were treated with 30 mM NAC for 2 h and then 1 mM H_2_O_2_ was added). After 30 min treated with H_2_O_2_ the cells were cultured in normal medium for 48 h. The apoptosis was evaluated by flow cytometry.

H_2_O_2_ has been shown to induce cell apoptosis, where p16 plays a crucial role^6^,^16^. Given a correlation between apoptosis and p16 phosphorylation was observed, we asked whether H_2_O_2_ could influence the phosphorylation of p16. To this end, we applied H_2_O_2_ in different doses (0.6, 0.8, 1, 1.2 and 1.4 mM) to 293T cells, in which the level of p16 phosphorylation is low (Data not shown). H_2_O_2_ from 0.6 to 1 mM induced cell apoptosis in a dose-dependent manner (Fig. 2A) and 1 mM H_2_O_2_ appeared to be the most effective dose for inducing cell apoptosis. Meanwhile, we observed that mitochondrial superoxide was increased and the mitochondria membrane potential was decreased in cells treated with H_2_O_2_ (Fig. 2B and C). A drastically increase of phosphorylation in endogenous p16 upon H_2_O_2_ treatment (Fig. 2D and Fig. S1 in the Supplementary Information) revealed H_2_O_2_ as an effective inducer of p16 phosphorylation. To substantiate the involvement of oxidative stress resulted from ROS generation in the H_2_O_2_-induced p16 phosphorylation, NAC (N-acetyl-L-cysteine, a general antioxidant) was used. The results from CoIP assays showed that NAC treatment significantly reduced H_2_O_2_-induced p16 phosphorylation and apoptosis (Fig. 2E and F, Fig. S2 in the Supplementary Information), suggesting that oxidative stress plays an essential role in the induction of p16 phosphorylation and apoptosis.

### Phosphorylated p16 is preferentially associated with CDK4

**Figure 3:**
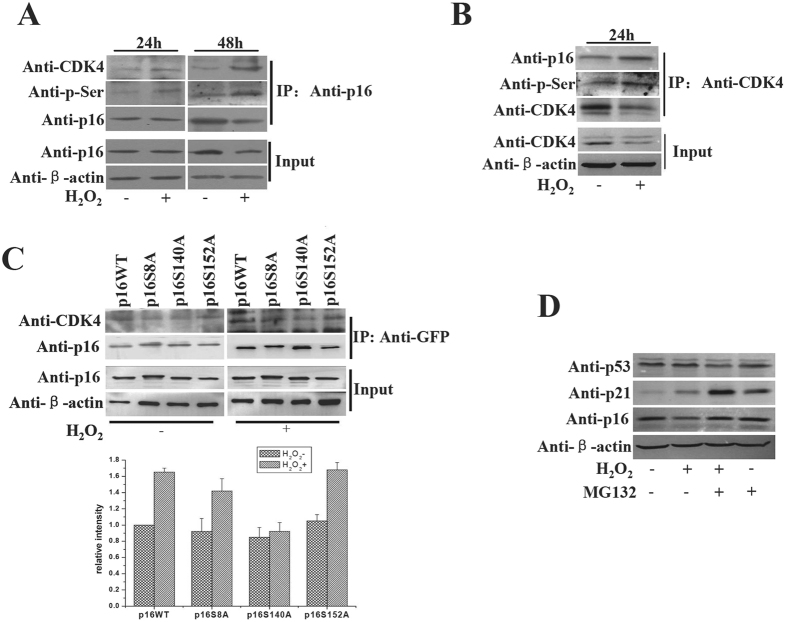
Phosphorylated p16 protein had an enhanced association with CDK4. 293T cells were treated with 1 mM H_2_O_2_ 24 h or 48 h. Whole-cell extracts were prepared and immunoprecipitated with anti-p16 antibody (**A**) or anti-CDK4 antibody (**B**). Precipitates were subjected to immunoblotting with anti-p16, anti-CDK4 and anti-phosphserine antibodies. (**C**) 293T cells were transfected with wild type p16-GFP or mutant p16-GFP expression plasmids, and after 24 h treated with 1 mM H_2_O_2_. After 24 h whole-cell extracts were prepared and immunoprecipitated with anti-GFP antibody. Precipitates were subjected to immunoblotting with anti-p16 and anti-CDK4 antibodies. Upper: The western of p16, CDK4 and actin as indicated. Lower: the results was photodensitometry analysis of the western bands from three experiments, and the results are presented as the relative intensity ratio between CDK4 bands and IP-p16 bands. (**D**) Western blot analysis of the p16, p21 and p53 proteins in 293T cells treated with 1 mM H_2_O_2_ or 20 μM MG132 or H_2_O_2_ together with MG132, then cultured 48 h.

Given that p16 function is tightly associated with CDK4, a key regulator of cell cycle progression, we next investigated whether the phosphorylation of p16 affected the interaction between p16 and CDK4. Upon H_2_O_2_ treatment for 24 or 48 h, more phosphorylated p16 was detected in the complex immunoprecipitated by anti-p16 antibody as expected (Fig. 3A and Fig. S3 in the Supplementary Information). Importantly, the level of CDK4 was increased significantly in cells stimulated with H_2_O_2_ as compared to that in untreated cells (Fig. 3A). A reciprocal immunoprecipitation by CDK4 confirmed that phosphorylated p16 was indeed increased in the p16-CDK4 complexes in response to H_2_O_2_ (Fig. 3B and Fig. S4 in the Supplementary Information). These data suggest that the hyper-phosphorylated p16 proteins possess upregulated CDK4-binding ability. However, the CoIP assays demonstrated that the p16S140A interacted with less CDK4 then wild type p16 without or with H_2_O_2_ treated (Fig. 3C and Fig. S8 in the Supplementary Information). Notably, the total p16 proteins were reduced upon the H_2_O_2_ treatment (Fig. 2D, E and 3A), indicating that p16 may be degraded after H_2_O_2_ treatment. In contrast to other critical cell cycle regulators, including p53 and p21, the level of p16 decreased by H_2_O_2_ treatment and restored in the presence of MG132, a widely used proteasome inhibitor (Fig. 3D and Fig. S5 in the Supplementary Information). Collectively, these data suggest that increased serine phosphorylation facilitates the association of CDK4 with p16 and the degradation of p16 can be triggered by H_2_O_2_.

### Ser 140 phosphorylation antagonizes with the Arg 138 methylation on p16

**Figure 4:**
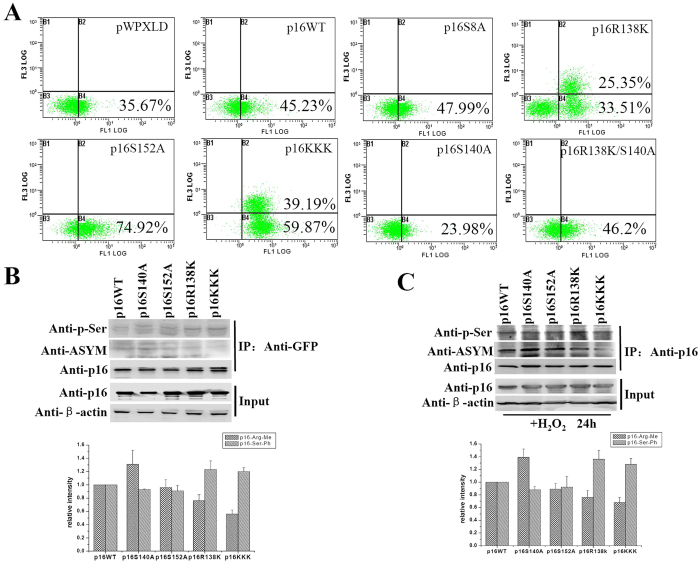
The antagonistic crosstalk between Ser 140 phosphorylation and Arg 138 methylation of p16 protein. (**A**) 293T cells were transfected with empty control vector (pWPXLD), wild type p16, or mutant p16 expression plasmids. After 24 h the cells were treated with 1 mM H_2_O_2_ for 30 min, and after 24 h the apoptosis was evaluated by flow cytometry. Apoptosis rates were calculated on the basis of 15 000 cells. (**B**,**C**) CoIP assays with anti-GFP or anti-p16 and detected with anti-p16, anti-ASYM or anti-phosphserine antibody. 293T cells transfected with wild type p16 or mutant p16 expression plasmids. After 24 h the cells were treated with 1 mM H_2_O_2_ for 30 min, and cultured 24 h before harvest. Upper: The western of p16, mehtylated-p16, phosphorylated-p16 and actin as indicated. Lower: The results were the photodensitometry analysis of the western bands from three experiments, and the results are presented as the relative intensity ratio between phosphorylation or methylation bands and IP-p16 bands.

To understand how arginine and serine modifications are coordinated to regulate p16 function in apoptosis, we ectopically overexpressed different p16 mutants in 293T cells and subsequently treated those cells with H_2_O_2_. As expected, H_2_O_2_ efficiently caused cell apoptosis (35.67%) (Fig. 4A), while only 5.66% apoptotic cells were scored in the control (Fig. 1C). Compared to wild type p16, arginine or serine mutants, especially p16R138K and p16KKK, enhanced cell apoptosis induced by H_2_O_2_ (Fig. 4A) In contrast, the expression of p16S140A mutant reduced apoptotic cells to 23.98% (Fig. 4A). Addionally, the percentage of apoptotic cells induced by p16S140A/R138K mutant was close to that of cells overexpressing wild type p16 (Fig. 4A), suggesting that the Ser 140 mutant might counteract the apoptosis induced by Arg 138 mutant. Next, we asked whether an interplay between methylation and phosphorylation existed on p16. Analysis of methylated and phosphorylated p16 mutants immunoprecipitated by anti-p16 antibody demonstrated that arginine methylation in p16S140A was increased as compare to that in wild type p16 (Fig. 4B and C, lane 2 compared with lane 1 in line 2) (Fig. S9 and S6 in the Supplementary Information). In parallel, serine phosphorylation in p16R138K and p16KKK were higher than that in wild type p16 with or without H_2_O_2_ treated (Fig. 4B and C, lane 4 compared with lane 1 in line 1). These results implicate that an antagonistic mechanism coordinates Arg 138 methylation with Ser 140 phosphorylation on p16.

### H_2_O_2_ induced cell senescence is accompanied by p16 phosphorylation

**Figure 5:**
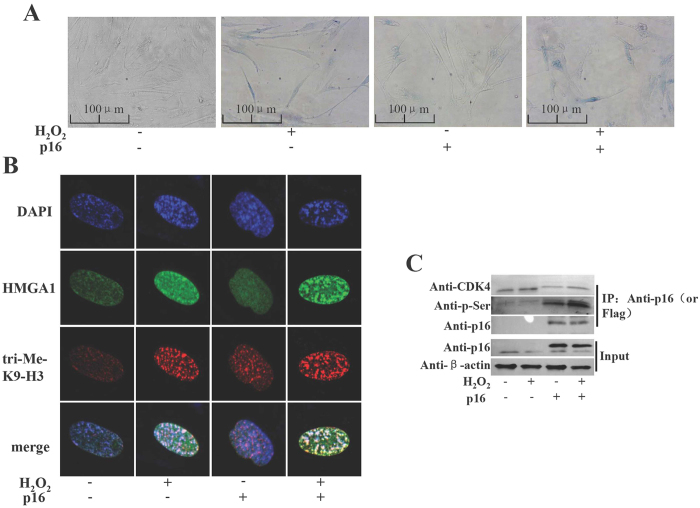
H_2_O_2_ induced the senescence and p16 phosphorylation in WI-38 cells. (**A**) WI-38 cells were transfected with empty control vector (pWPXLD), or wild type p16 expression plasmids. After 24 h the cells were treated with 1 mM H_2_O_2_ for 30 min and then cultured 3 days. The cells were treated with 1 mM H_2_O_2_ for 30 min again. After 3 days representative photomicrographs of the SA-β-gal staining are detected under a microscope. (**B**) Cells were stained with DAPI, and the heterochromatic foci were visualized by fluorescence microscopy. The 3meK9H3 was immunostained in red, and HMGA1 in green. The nuclei were counterstained with DAPI (blue). The cells were visualized under a confocal microscope. (**C**) the WI-38 cells transfected with plasmids indicated and treated with H_2_O_2_ as described above. CoIP with anti-p16 or anti-Flag, and detected with anti-CDK4, anti-p16 or anti-phosphserine antibody. Input: proteins prior to immunoprecipitation.

The critical role of p16 in cell senescence has been well established^17^. We thus decided to examine the H_2_O_2_-dependent p16 function in cell senescence. We found that p16 overexpression or H_2_O_2_ treatment alone induced senescence in WI-38 cells and the combination of both resulted in an additive effect (Fig. 5A). Meanwhile, the senescence-associated heterochromatin foci (SAHF) assay confirmed the observations in senescence cell staining. Both 3MeK9H3 and HMGA1, two classic markers of SAHF, localized to the specific heterochromatic foci in cells transfected with p16 and treated with H_2_O_2_ (Fig. 5B). The colocalization of them in discrete foci became more obvious when p16 overexpression and H_2_O_2_ treatment were applied together (Fig. 5B). These results indicate that overexpression of p16 and H_2_O_2_ treatment can induce WI-38 cell senescence. Consistent with the observations in 293T cells, our CoIP experiments showed that the level of p16 phosphorylation and the association of CDK4 with p16 increased upon the H_2_O_2_ treatment in WI-38 cells (Fig. 5C and Fig. S7 in the Supplementary Information). Taken together, these data suggest an important role of H_2_O_2_-induced p16 phosphorylation in cell senescence.

## Discussion

p16 is well known as a negative regulator of the cell cycle and aberrantly expressed in various malignancies^18^. Previous studies have shown that the inactivation of p16 is mediated through distinct mechanisms, including DNA mutations^19^,^20^ and methylation of promoters^21^. A growing number of PTMs have been identified on p16^9^,^10^,^11^,^12^. However, their functions in the control of p16 during cell apoptosis and senescence remain largely unclear. A previous study showed that both expression and phosphorylation level of p16 were elevated in aging prostate cells, and the phosphorylated p16 exhibited an increased binding affinity to CDK4/6^9^. Subsequently, Ser 7, 8, 140 and 152 of p16 have been identified as phosphorylation sites in WI-38 cells. The phosphorylation of Ser 152 appeared to be important for binding of CDK4 to p16, whereas Ser 8 phosphorylation impaired this interaction^10^,^11^. In this study, we showed p16S8A and p16S140A mutants relieved the cell cycle arrest and apoptosis induced by p16. In contrast, p16S152A mutant led to an opposite outcome (Fig. 1B and C). These observations imply that the phosphorylation of different residues may have different physiological functions. Furthermore we have previously shown that specific arginine residues of p16 protein are methylated by PRMT6, resulting in impaired p16-CDK4 association as well as alleviated cell cycle arrest at G1^12^. Therefore, distinct PTMs on p16 may control p16 function in different manners.

It has been shown that the arginine residues of FOXO1 and BAD were methylated by PRMT1, and the methylation in turn blocked Akt-mediated serine phosphorylation^13^,^14^. Another recent study demonstrated that Arg 1175 methylation increased trans-autophosphorylation at Tyr 1173 of EGFR^15^. These evidence suggest that crosstalk between arginine methylation and serine phosphorylation may be crucial for the control of protein functions. Here, we found that p16R138K overexpression in 293T cells was associated with an increase of G1 cells, while the overexpression of p16S140A led to a reduction of G1 cells. When cells were transfected with p16S140A/R138K, the population of G1 cells was largely unaffected as compared to that in cells overexpressingwild type p16 (Fig. 1B). Moreover, arginine methylation of p16 was upregulated when S140 was mutated to alanine. We also observed elevated serine phosphorylation of p16 as R138 was replaced by a lysine (Fig. 4B and C). Consistent with previous reports^13^,^14^, these results suggest an antagonistic effect existing between Arg 138 methylation and Ser 140 phosphorylation on p16.

Oxidative stress is involved in the death of neurons and leads to the apoptosis in the neurological diseases^22^. ROS may be the first-stage initiators inducing neuron apoptosis or act as a signal in apoptotic cascade^23^,^24^. As a typical ROS, hydrogen peroxide (H_2_O_2_) is widely used as an apoptosis inducer^16^. It has been shown that H_2_O_2_ induced DNA damage and apoptosis in human neuroblastoma SK-N-MC cells^25^. H_2_O_2_ could also elicit Ca^2+^ influx through store-operated channels (SOCs) in HEK-293 cells^26^. Moreover, p16 level was rapidly upregulated in a p38 stress-activated protein kinase-dependent manner upon H_2_O_2_-induced oxidative stress^27^. It has also been demonstrated that the H_2_O_2_-induced senescence in NHEKs and HepG2 cells is due to the enhanced p16 expression mediated by suppressed methylation on p16 promoter^28^,^29^. These data suggest that different mechanisms associated with p16 function may be involved in H_2_O_2_-induce cell senescence and apoptosis. Here we found that H_2_O_2_ induced cell apoptosis in a dose-dependent manner (Fig. 2A). Endogenous p16 was substantially phosphorylated upon H_2_O_2_ treatment (Fig. 2D) and NAC reduced the H_2_O_2_-induced phosphorylation of p16 (Fig. 2E). Importantly, we provided evidence that the phosphorylation of p16 improved the binding affinity of p16 with CDK4 (Fig. 3A). These results suggest that H_2_O_2_ enhances the phosphorylation of p16 protein, which in turn influences cell cycle as well as apoptosis. In addition, we found that H_2_O_2_ effectively promoted p16 phosphorylation and cell senescence in WI-38 (Fig. 5A,B and C). We thus speculate that the phosphorylation of p16 promoted is involved in H_2_O_2_-induced cell apoptosis and senescence. p16 has previously been shown to have a relatively short half-life ranging from 30 min to 3.5 h in a variety of cancer cell lines^30^. A direct interaction with REGγ could also facilitate p16 degradation^31^. p16 can probably be subjected to ubiquitination-mediated proteasomal degradation when it is phosphorylated^32^. In agree with this observation, we showed that the p16 level was reduced in response to H_2_O_2_ treatment and restored by the proteasome inhibitor MG132 (Fig. 3D). This suggests that H_2_O_2_ is able to promote the degradation of p16 proteins.

**Figure 6:**
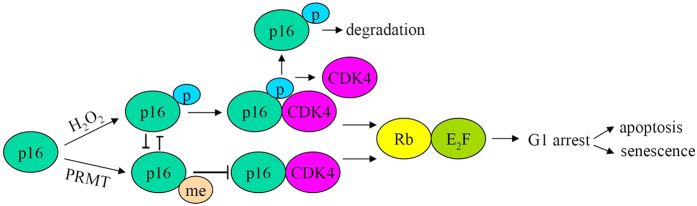
Model for phosphorylation and degradation of p16 induced by H_2_O_2_. Firstly, H_2_O_2_ induced phosphorylation of p16, and then the phosphorylated p16 interacted with CDK4. p16-CDK4 facilitate the stabilization of Rb-E_2_F_1_, thereby leading to the cell cycle arrest at G1 phase. Subsequently the phosphorylated-p16 was degraded. However, the methylated p16 decrease the formation of p16-CDK4 complexes. Arrows and bars represent positive and negative regulation, respectively. Me denotes methylation; P denotes phosphorylated.

In summary, we have provided evidence that H_2_O_2_ induces the phosphorylation of p16 protein, which enhances CDK4 association. Meanwhile, the phosphorylated p16 is degraded probably by ubiquitination-mediated proteasomal system (Fig. 6). Furthermore, our data support an antagonistic crosstalk between the Ser 140 phosphorylation and the Arg 138 methylation on p16. These findings will pave the way to future therapeutic treatment associated with functional targeting of p16.

## Methods

### Cell culture and transient transfection

The cell lines (293T and WI-38) were cultured in DMEM supplemented with 10% FBS (fetal bovine serum), 100 U/ml penicillin and 100 μg/ml streptomycin, and kept in a humidified atmosphere containing 5% CO_2_ at 37 °C. Cells were incubated in culture medium with or without H_2_O_2_ (0.6 mM, 0.8 mM, 1 mM, 1.2 mM or 1.4 mM) for 30 min, and then cultured 24 h or 48 h in the medium without H_2_O_2_. The transfection of WI-38 and 293T cells was carried out using the Lipofectamine 2000 reagent (Invitrogen) or the PEI reagent (Sigma).

### Plasmids

The p16-EGFP-N1 plasmid was provided by Dr. Jun Chen (New York Medical College, USA). The specific site mutations of the arginine or serine residues were introduced into the p16 cDNA region by using a two-step PCR procedure. Two simultaneous PCR reactions, using p16-EGFP-N1 as template, were performed. Amplified fragments from each PCR reaction were purified, mixed, and subjected to a second round of PCR using two external primers. The mutagenic sequence for the Ser 8 residue was AGC (Serine) to GCC (Alanine), for the Ser 140 residue was AGT (Serine) to GCT (Alanine), and for the Ser 152 residue was TCA (Serine) to GCA (Alanine). The amplified PCR products were inserted into the *Hind*III and *BamH*I sites of EGFP-N1 vector, and the correct insertion was verified by DNA sequencing. The arginine residue mutations of p16 vectors were described previously^12^. Then, these p16 cDNAs including specific site mutations of the arginine or serine residues, were amplified using PCR reaction and the Flag tag were introduced into the PCR products, which were inserted into the *Pme*I and *Spe*I sites of pWPXLD vector.

### Western blotting and co-immunoprecipitation (CoIP)

Endogenous expression of p16, p21 and p53 proteins was detected by Western blotting. 293T cells were treated with 30 mM NAC (antioxidant N-acetyl-L-cysteine) for 2 h and then 1 mM H_2_O_2_ was added. After 30 min the cells were cultured in normal medium for 48 h. 1 × 10^6^ cells were digested and lysed in lysis buffer (50 mM Tris-HCl, pH 8.0; 150 mM NaCl; 0.5% NP-40; 1 mM EDTA; protease inhibitors cocktail) for 30 min at 4 °C after they were washed twice with PBS buffer. Total cell extracts were separated in 15% SDS-polyacrylamide gel electrophoresis (PAGE), then transferred to a polyvinylidene fluoride membrane. The membrane was incubated with anti-p53 (Sigma, p6495), anti-p16 (BD, 551154), anti-21 (Santa cruz, sc-756), or anti-β-actin (Sigma, A1978) antibodies, and visualized by using the Chemiluminescent Substrate method with the SuperSignal West Pico kit provided by Pierce Co. β-actin was used as an internal control for normalizing the loading materials.

Co-precipitation of p16 with CDK4 was performed in 293T cells. Cells were lysed in lysis buffer (50 mM Tris-HCl, pH 8.0; 150 mM NaCl; 0.5% NP-40; 1 mM EDTA; protease inhibitor cocktail). Total cell extracts were incubated with the anti-GFP (Abcam, ab1218), anti-CDK4 (Santa Cruz, sc-260) and anti-p16 antibodies, with gentle shaking for 1 h at 4 °C, followed by the addition of 40 μl of Protein A-agarose and an incubation for another 3 h. The pellets were collected and washed twice with Buffer A (20 mM Tris-HCl, pH 8.0; 10 mM NaCl; 0.5% NP-40; 1 mM EDTA). The beads were resuspended in 50 μl of 5× loading buffer and boiled for 10 min. The proteins were separated in a 12% or 15% SDS-PAGE gel and then transferred to polyvinylidene fluoride membrane for immunoblotting detection with anti-p16, anti-CDK4, anti-phosphserine (Millipore, ab1603) or anti-dimethyl-arginine (asymmetric) (Millipore, 07-414, ASYM24) antibodies.

### Flow cytometric analysis

293T cells were trypsinized and washed with cold PBS once and then fixed in 70% ethanol and stored at 4 °C for 30 min. Fixed cells were washed with PBS and suspended in 100 μl of PBS, added with 1 μl 10 mg/ml RNAaseA and 100 μl propidium iodide. Stained cells were incubated at room temperature for 30 min in the dark. The cellular DNA content was quantified by flow cytometric analysis using a FACS Calibur (Beckman coulter, USA). The apoptosis assay was performed by using the annexin V–fluorescein isothiocyanate (FITC) apoptosis detection kit (Nanjingkaiji, Nanjing, China), and detected by flow cytometry (excitation at 488 nm; emission at 530 nm) with an FITC signal detector, and propidium iodide (PI) staining with a phycoerythrin emission signal detector.

### Senescence-associated galactosidase activity assay

WI-38 cells were transfected with the p16 expression vectors. After 24 h, the cells were treated with 1 mM H_2_O_2_ for 30 min, and then cultured in normal medium. At day 3 after treatment, cells were treated with H_2_O_2_ for another 3 days. Cells were then processed using a Senescence β-Galactosidase Staining Kit (Cell Signaling Technology), before they were examined under a light microscope (Nikon, Japan) and the images were collected at ×20 magnification with appropriate filters. These experiments were repeated three times, and one of the representative results is shown.

### Immunofluorescence staining

WI-38 cells were transfected with the p16 expression vectors. After 24 h, the cells were treated with 1 mM H_2_O_2_ for 30 min, and then cultured in the normal medium. At day 3 after treatment, cells were treated with H_2_O_2_ for another 3 days. The treated WI-38 cells were washed twice in PBS, fixed with 4% paraformaldehyde for 15 min, permeabilized with 0.2% Triton X-100 at room temperature and then quenched in ice-cold PBS. Trimethylated histone H3 was detected with anti-3meK9H3 antibody (Mollipore, 07-523) and visualized with a TRITC-conjugated anti-rabbit IgG secondary antibody (Zhongshan, China), and then incubated with anti-HMGA1 (Sigma, SAB1401183) antibody serum for 1 h and visualized with a FITC-conjugated secondary antibody. Finally, cells were stained with DAPI before they were visualized under an Olympus FV1000 (Olympus, Japan) confocal microscope.

### Mitochondrial ROS assay

293T cells were trypsinized and washed with PBS once and then incubate with 1 ml 5 μM MitoSOX^TM^ reagent (M36008, invitrogen) for 10 min at 37 °C in the dark. Then wash cells gently three times with PBS, and detected by flow cytometry (excitation at 488 nm; emission at 580 nm).

### Mitochondria membrane potential (ΔΨm) assay

Mitochondria membrane potential was determined with JC-1 (Beyotime Biotech, Nantong, China). Briefly, 293T cells were seeded in 60 mm plates. After experimental treatment, cells were trypsinized and washed with PBS once, and then added with 1 ml staining dye and incubated at 37 °C for 20 min. After this, cells were washed twice with cold JC-1 staining buffer (1×), and detected by flow cytometry (excitation at 488 nm; emission at 529 nm).

## Additional Information

**How to cite this article**: Lu, Y. *et al*. The interplay between p16 serine phosphorylation and arginine methylation determines its function in modulating cellular apoptosis and senescence. *Sci. Rep.* **7**, 41390; doi: 10.1038/srep41390 (2017).

**Publisher's note:** Springer Nature remains neutral with regard to jurisdictional claims in published maps and institutional affiliations.

## Supplementary Material

Supplementary Information
