# Supplementary material for: The interplay between p16 serine phosphorylation and arginine methylation determines its function in modulating cellular apoptosis and senescence
Source: Sci Rep. 2017 Jan 25;7:41390. doi: 10.1038/srep41390 (PMC5264599; doi:10.1038/srep41390)
Supplement: Supplementary Information [file srep41390-s1.doc]

The interplay between p16 serine phosphorylation and arginine methylation determines its function in modulating cellular apoptosis and senescence

Yang Lu^1^, Wenlong Ma^1^, Zhongwei Li^1^, Jun Lu^1^, Xiuli Wang^1,2*^

^1^ Institute of Genetics and Cytology, Northeast Normal University; ^2^School of Life Sciences, Northeast Normal University, Changchun 130024, P. R. China. ^*^Correspondence and requests for materials should be addressed to X. Wang (email: [wangxl034@nenu.edu.cn](mailto:wangxl034@nenu.edu.cn)). Tel./Fax.: +86 43185099607.

Suppl. Fig.1


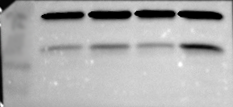

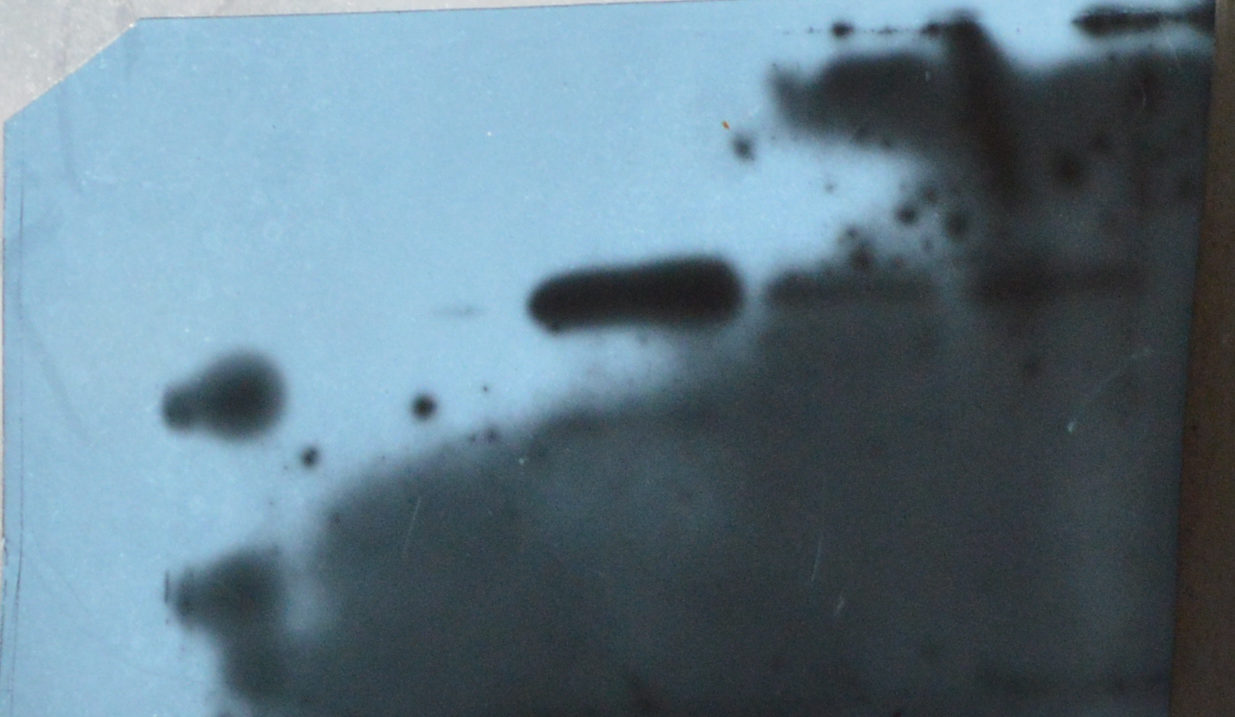


p16-p-Ser


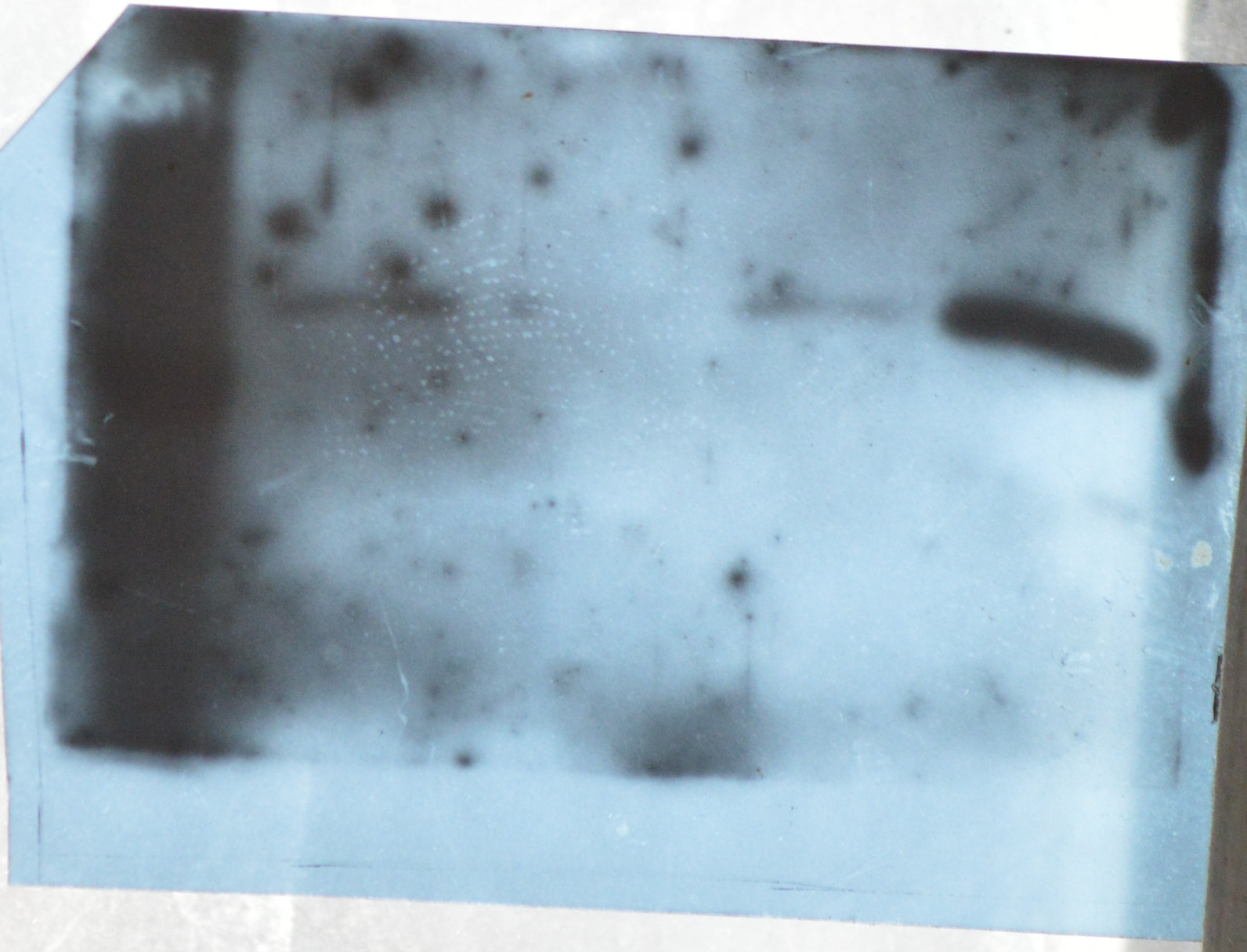


p16-p-Ser

p16-p-Ser

-H_2_O_2_

+H_2_O_2_

-H_2_O_2_

+H_2_O_2_

24h

48h


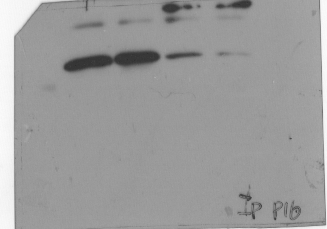


p16

-H_2_O_2_

+H_2_O_2_

-H_2_O_2_

+H_2_O_2_

24h

48h


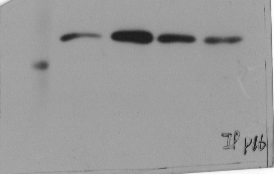


p16


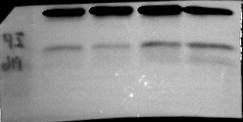


p16

Suppl. Fig.1. H_2_O_2_ induced serine phosphorylation of p16. 293T cell treated 30 min by 1mM H_2_O_2_, and then cultured for 24 h or 48 h. Cell extracts were prepared and precipitated with anti-p16 antibody, then detected with anti-p16 antibody or anti-phosphserine antibody. These are the results of three experiments.

Suppl. Fig.2


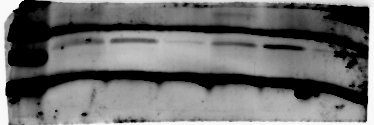

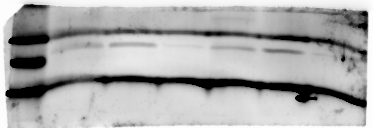


p16-p-Ser

p16-p-Ser

24h

48h

H_2_O_2_

NAC

-

-

+

-

+

+

-

-

+

-

+

+


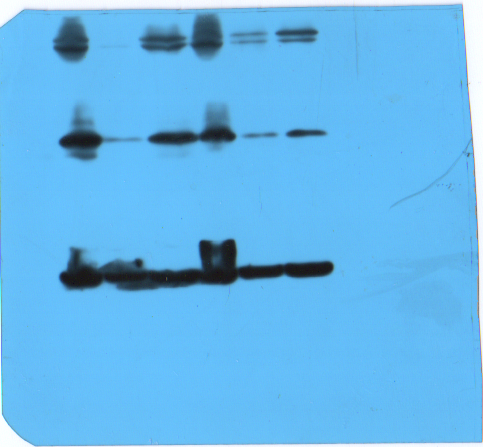


p16


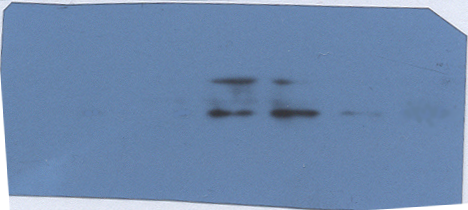


48h

-

-

+

-

+

+

H_2_O_2_

NAC

p16-p-Ser


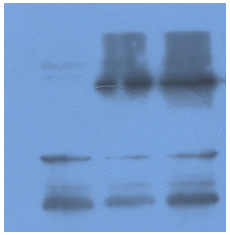


p16

Suppl. Fig.2. The 293T cells were pre-treated with 20 mM N-acetyl-L-cysteine (NAC), a general antioxidant, for 2 h and then treated the cells with 1 mM H_2_O_2_ for 48 h. The cell extracts were prepared and precipitated with anti-p16 antibody, then detected in immunoblotting with anti-p16 antibody or anti-phosphserine antibody. These are the results of two experiments. The below two results are different exposure time.

Suppl. Fig.3

CDK4


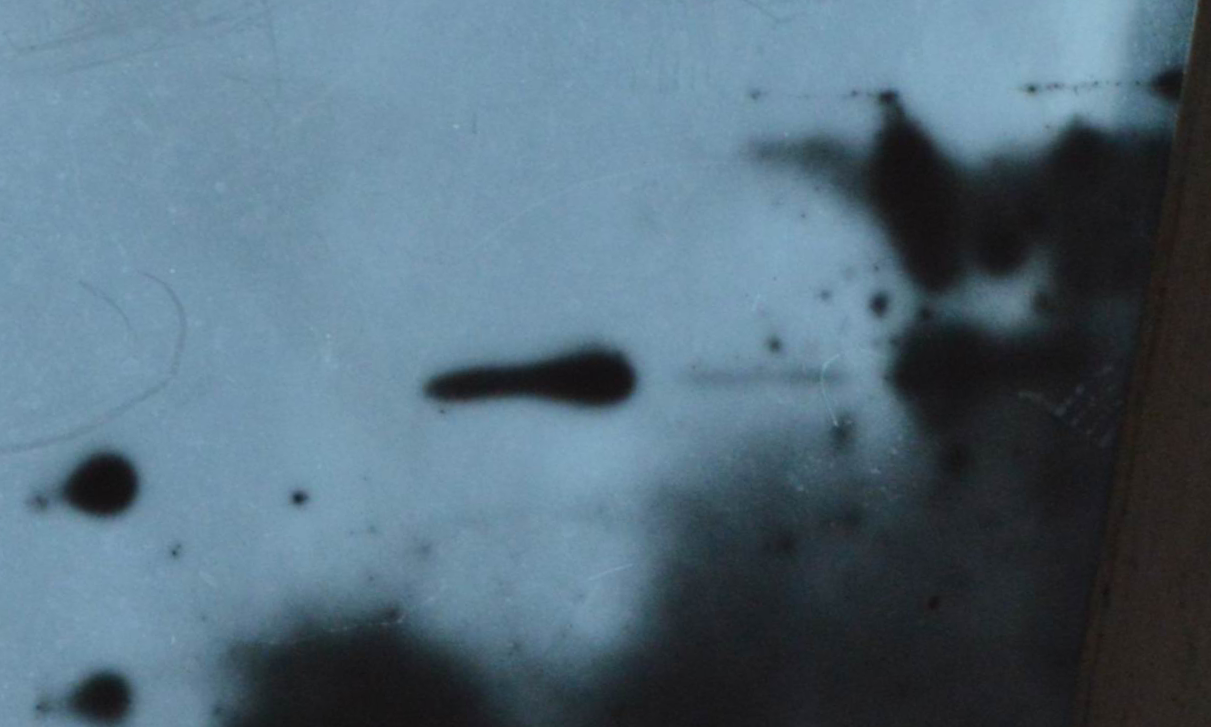

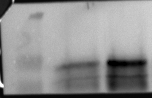

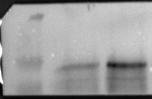


CDK4

p16-p-Ser

- H_2_O_2_

+ H_2_O_2_

24 h


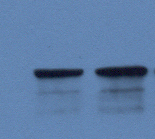


p16-p-Ser


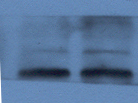


CDK4


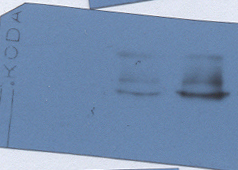


CDK4


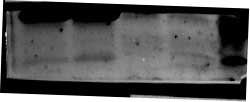


p16-p-Ser


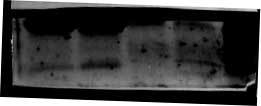


p16-p-Ser

- H_2_O_2_

+ H_2_O_2_

48h

Suppl. Fig. 3. Phosphorylated p16 protein had an enhanced association with CDK4. 293T cells were treated with 1 mM H_2_O_2_ 24 h or 48 h. Whole-cell extracts were prepared and immunoprecipitated with anti-p16 antibody. Precipitates were subjected to immunoblotting with anti-p16, anti-CDK4 and anti-phosphserine antibodies. The left results are from two experiments. The below two results (left and right, respectively) are different exposure time.

Suppl. Fig.4

p16-p-Ser


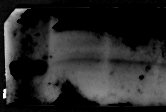

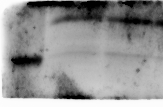


p16-p-Ser

- H_2_O_2_

+ H_2_O_2_

24 h


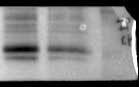


CDK4


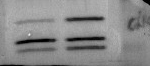


p16

- H_2_O_2_

+ H_2_O_2_

24 h

Suppl. Fig.4. p16 protein associated with CDK4 had an enhanced phosphorylation. 293T cells were treated with 1 mM H_2_O_2_ 24 h. The cell extracts were prepared and immunoprecipitated with anti-CDK4 antibody. Precipitates were subjected to immunoblotting with anti-p16, anti-CDK4 and anti-phosphserine antibodies. The left two results are different exposure time.

Suppl. Fig.5


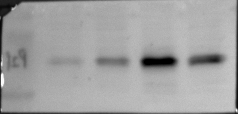


p21


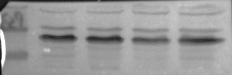


p53

p16


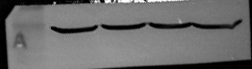


actin


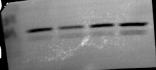


H_2_O_2_

MG132

-

-

+

-

+

+

-

+

H_2_O_2_

-

-

+

-

+

+

-

+

Suppl. Fig.5. Western blot analysis of the p16, p21 and p53 proteins in 293T cells. The cells were treated with 1mM H_2_O_2_ or 20μM MG132 or H_2_O_2_ together with MG132, then cultured 48h. the whole-cell extracts were prepared and detected with anti-p16, anti-p21 and anti-p53 antibodies

Suppl. Fig.6


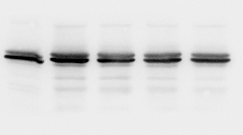

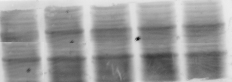

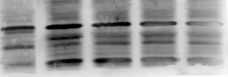


IP：anti-p16

p16

p16-Me-Arg

p16-p-Ser


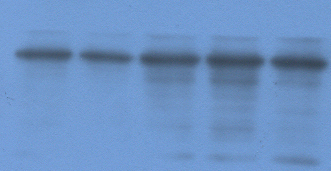

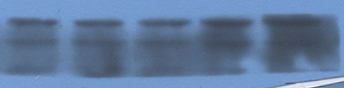

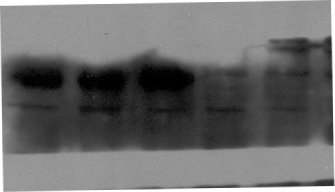


Suppl. Fig. 6. 293T cells transfected with wild type p16 or mutant p16 expression plasmids. After 24 h the cells were treated with 1 mM H_2_O_2_ for 30 min, and cultured 24 h before harvest. CoIP assays with anti-p16 and detected with anti-p16, anti-ASYM or anti-phosphserine antibodies. These are the results of two experiments.

Suppl. Fig.7

CDK4


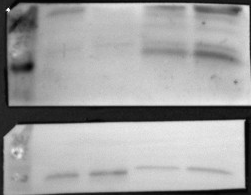


p16-p-Ser


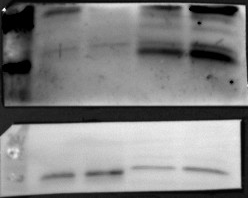


CDK4

p16-p-Ser

H_2_O_2_

-

-

+

-

+

+

-

+

p16

Suppl. Fig.7. WI-38 cells were transfected with empty control vector (pWPXLD), or wild type p16 expression plasmids. After 24 h the cells were treated with 1 mM H_2_O_2_ for 30 min and then cultured 3 days. CoIP with anti-p16 or anti-Flag, and detected with anti-CDK4, anti-p16 or anti-phosphserine antibody. The results are different exposure time.

Suppl. Fig.8


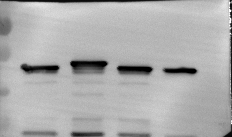

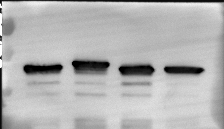


CDK4

CDK4

p16

CDK4

CDK4

p16


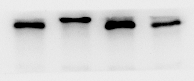

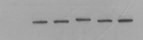


p16

p16

-H_2_O_2_

+H_2_O_2_

IP: anti-GFP


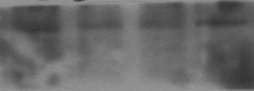

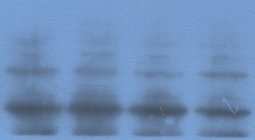

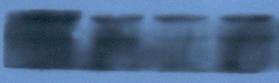

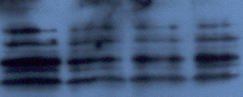


Suppl. Fig. 8. The mutants of p16 protein associated with CDK4. 293T cells were transfected with wild type p16 or mutant p16 expression plasmids. After 24 h, the cells treated with H_2_O_2_. Whole-cell extracts were prepared and immunoprecipitated with anti-GFP antibody. Precipitates were subjected to immunoblotting with anti-p16 and anti-CDK4 antibodies. The results are from two experiments.

Suppl. Fig.9


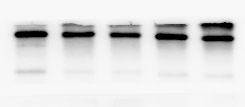

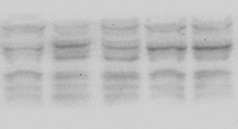

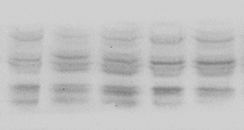

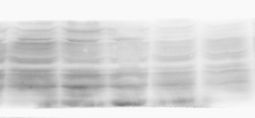

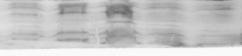


p16

p16-Me-Arg

p16-p-Ser

IP: anti-GFP


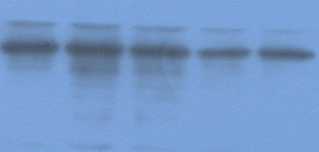


Suppl. Fig. 9. 293T cells transfected with wild type p16 or mutant p16 expression plasmids. After 48 h, CoIP assays with anti-GFP and detected with anti-p16, anti-ASYM or anti-phosphserine antibodies. These are the results of two experiments.
